# Pain Catastrophizing and Functional Activation During Occlusion in TMD Patients—An Interventional Study

**DOI:** 10.1002/hbm.70051

**Published:** 2024-10-19

**Authors:** K. Klepzig, M. Domin, B. Kordass, M. Lotze

**Affiliations:** ^1^ Functional Imaging Unit, Center of Diagnostic Radiology and Neuroradiology University Medicine Greifswald Greifswald Germany; ^2^ Department of Dental Radiology, Centre of Dentistry and Oral Health University Medicine Greifswald Greifswald Germany; ^3^ Department of Clinical Dental CAD/CAM and CMD‐Treatment, Centre of Dentistry and Oral Health University Medicine Greifswald Greifswald Germany

**Keywords:** chronic pain, craniomandibular disorder (CMD), fMRI, interventional trial, Michigan splint, pain catastrophizing, temporomandibular disorder (TMD)

## Abstract

In temporomandibular disorder (TMD), the effects of standard interventions such as using an occlusal splint and its impact on pain relief and pain catastrophizing are poorly understood. Earlier work pointed to a crucial role of insula activation with changes in pain relief by occlusal splint treatment. We performed a functional imaging study using specially developed splint systems to allow for a placebo‐controlled longitudinal design. Using functional MRI we examined 20 TMD patients during repetitive occlusal movements at baseline and over the course of splint therapy and also collected self‐reported pain catastrophizing. For balancing performance between baseline and after intervention we used occlusion force measures in an individualized fMRI‐splint system. Splint therapy lasted for approximately 7 weeks with one group selected by randomization wearing a palatine placebo splint over the first 3 weeks (delayed start; 11 individuals). As expected, fMRI activation in areas involved in pain processing (insula, primary and secondary somatosensory cortex) decreased with intervention. At baseline a positive correlation between activation of the left anterior insula and pain catastrophizing was present. Both parameters decreased over intervention while associations were primarily observable for patients with rather mild TMD.

## Graphical abstract

The top illustration shows the longitudinal design of the complete interventional study.

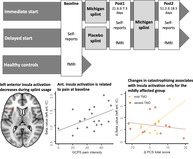

The bottom shows anterior left insula activation, which decreases by the intervention (left), is associated with pain intensity (middle), and is related to pain catastrophizing only for the mildly affected participants.

## 1 Introduction

Temporomandibular disorder (TMD) is a chronic pain disorder predominantly located around the mandibular joint extending into the forehead and neck (Gesch et al. 2004; Schindler et al. 2014). The occurrence and intensity of pain attacks are related to a number of different factors like stress on the mandibular joint, but also psychological distress such as anxiety (Dammann et al. 2020) and depression (Klepzig, Kordass, and Lotze 2024).

A standard treatment intervention is an individually fitted maxillary or mandibular splint which should be used as often as possible or only during nighttime (Ommerborn et al. 2010). In fact, splint therapies were shown to reduce pain intensity (Lickteig, Lotze, and Kordass 2013), to lower psychological distress (Dammann et al. 2020) and to decrease stress hormone concentration in TMD patients (Klepzig, Kordass, and Lotze 2024).

Functional MRI had been applied for investigating specific representation patterns of occlusal movements (Mihai, von Bohlen und Halbach, and Lotze 2013), and showed characteristic effects when applying occlusal splints (Ernst et al. 2019; Lickteig, Lotze, and Kordass 2013). Interestingly, these changes were associated with reductions in pain intensities (Ernst et al. 2019; Lickteig, Lotze, and Kordass 2013) and anxiety (Dammann et al. 2020), suggesting that changes in brain functioning are critical for the therapeutic effect of splint interventions. Pain catastrophizing (PC), a multidimensional construct comprising rumination, helplessness and pessimism, was found to be critical for the development of TMD (Park et al. 2015; Willassen et al. 2020) while such a way of thinking means to focus excessively on pain and to overestimate its impact on one's life (Sullivan et al. 2001). Interestingly, besides improvements in pain intensity, splint therapies can also reduce PC (Costa et al. 2015; Klepzig, Kordass, and Lotze 2024).

Therefore, in this study, we intended to examine associations between self‐reported PC and activations of relevant brain structures during occlusion over the course of splint therapy in TMD patients at three time points. We hypothesised that changes in PC during intervention would be accompanied by fMRI activation changes in areas relevant for pain processing (insula, the primary and secondary somatosensory cortex [S1, S2], anterior cingulate cortex [ACC] (Apkarian et al. 2005; Wager et al. 2013)). On the other hand, we expected that fMRI activation magnitude in motor areas of occlusal movements (primary motor cortex [M1], supplementary motor areas [SMA], superior parietal lobe [SPL] and intraparietal sulcus [IPS], anterior cerebellar hemispheres (Mihai, von Bohlen und Halbach, and Lotze 2013)) would increase over intervention. At baseline self‐reported distress would be positively associated with activation in the latter structures with a focus on the anterior insula and the ACC (Galambos et al. 2019). Effects of the intervention on pain intensities and stress‐related variables (self‐reports, salivary measures) in the same patient sample have been reported recently (Klepzig, Kordass, and Lotze 2024).

## 2 Materials and Methods

### 2.1 Participants

The *Centre of Dentistry and Oral Health* (diagnoses were obtained for each participant by B.K.) recruited 28 patients (27 women) aged between 18 and 72 years diagnosed with TMD on the basis of the research diagnostic criteria for TMDs (RDC) (Schiffman et al. 2010). Mostly women were included since women are typically more affected than men (Macfarlane et al. 2009). Data inclusion and exclusion are depicted in a flow chart (Figure S1). The inclusion criteria were age 18 years or older, pain in the temporomandibular joint area and surrounding muscles persisting for at least 6 months, occurring on at least 4 days a week. Finally, data of 20 female patients were considered for analyses (for details see Table 1). Eleven female controls (HCs; no chronic pain, no neurological and psychiatric disease) were recruited via social media for comparing self‐reported distress at baseline (Table 1). Two patients reported to use pain medication (nonsteroidal anti‐inflammatory drugs).

**TABLE 1:** Demographic details and self‐report data of patients and HCs at baseline.

	Healthy controls	TMD patients	Statistics
*N*	11	20	
Age (years)	28.4 ± 10.6	36.3 ± 13.7	*p* = 0.107
Sex (F:M)	11:0	20:0	
Handedness (L:R:A)	1:9:1	0:19:1	*p* = 0.436
Pain intensity (rest)	0.2 ± 0.5	16.5 ± 16.0	*p* < 0.001
Pain intensity (move)	0.7 ± 1.5	28.5 ± 25.4	*p* < 0.001
GCPS grade (0:I:II:III:IV)	2:9:0:0:0	1:7:7:1:3	*p* = 0.016
GCPS pain intensity	14.8 ± 9.4	45.3 ± 19.5	*p* < 0.001
PCS total score	10.0 ± 10.1	17.4 ± 10.8	*p* = 0.038

*Note:* Absolute numbers (*N*, Sex, Handedness, GCPS grade) and mean values with standard deviations are shown.

Abbreviations: A = ambidextrous; F = female; GCPS = Graded Chronic Pain Scale; L = left; M = male; move = during movement; PCS = Pain Catastrophizing Scale; R = right.

### 2.2 Study Design

**FIGURE 1:**
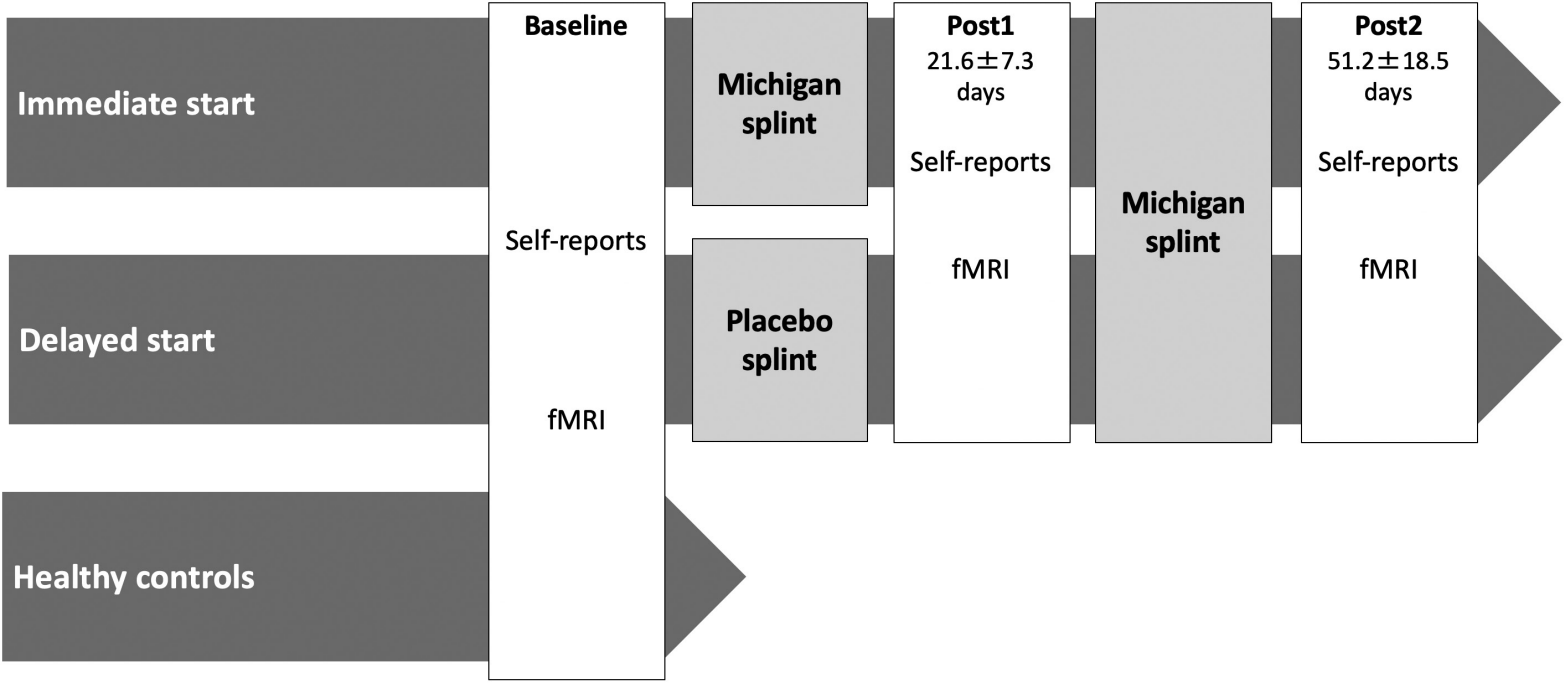
Study design.

In this placebo‐controlled study with a delayed start design TMD patients were examined three times: at baseline (before the onset of therapy), post1 (after 21.6 ± 7.3 days) and post2 (after 51.2 ± 18.5 days) (Figure 1). Originally, the two treatment periods were planned to last equally (3 weeks). However, short‐term postponements were relatively frequent for the third examination day probably due to other appointments of the patients which were not fixed at the beginning of the examination. Patients either received therapy with a Michigan splint over the entire period (immediate start, *n* = 9) or started with a palatine splint with no occlusal relevance first (therapy onset to post1) before wearing a Michigan splint (delayed start, *n* = 11) while they were randomly assigned to these treatment groups. According to the patients' loggings (completely available for 15 patients) the average wearing time per day considering the first 6 weeks after therapy onset did not differ between the two groups (9.8 and 10.2 h; *p* = 0.821), suggesting comparable therapy compliance. The delayed start approach was chosen due to ethical considerations (Michiels et al. 2018). HCs were only examined once. The trial was registered before starting the study (see trial DRKS00011931) and was approved by the Ethics Committee of the University Medicine Greifswald (BB 151/15). All participants provided written informed consent and were financially compensated.

### 2.3 Splint Intervention

A detailed description of the intervention can be found in our previous manuscript about this study (Klepzig, Kordass, and Lotze 2024). Splints were custom‐made by the Centre of Dentistry under the supervision of one member of the study team (BK). The Michigan splints were fabricated as maxillary splints using the semi‐custom articulator (Protar 5) after mounting the model with a face bow and centric registration (Michigan type). The placebo splint was adapted to the palatine and showed no occlusal contact. Participants were instructed to use the splint all day as often as possible, with the exception of ingestion. Therapy compliance was assessed via self‐reports of daily splint usage (in hours) and means of splint usage were computed for each week of therapy.

### 2.4 Self‐Reports of Pain Intensities and Perceived Stress

At the beginning of each examination, participants reported pain intensities at rest and after five occlusal movements using a pen and a visual analogue scale (VAS; 10 cm length) which represents pain intensities between “no pain” and “worst pain.” Pain intensities were scored as the distance between the left beginning of the scale and the mark in mm. Also, participants completed the pain catastrophizing scale (PCS) (Sullivan, Bishop, and Pivik 1995). To classify participants regarding pain severity they also completed the graded chronic pain status (GCPS) (Von Korff et al. 1992) at baseline. We computed pain intensity and the GCPS grade (Von Korff et al. 1992) with values below 2 indicating mild TMD and values above 1 indicating severe TMD, hence, obtaining two similarly sized groups (*n* = 8, *n* = 11). One patient provided only incomplete data (GCPS, PCS) and could not be considered for corresponding analyses.

### 2.5 Occlusal Movement Paradigm (fMRI)

The examination consisted of three tasks. During the “tube task,” patients were instructed to close and open their jaw in a relaxed manner at a frequency of 1 Hz while wearing a splint equipped with a tube (Heidelberger extension line, B. Braun Melsungen AG, Melsungen, Germany) which was connected to a pressure transducer and a Varioport Portable Recorder (Becker Meditec, Karlsruhe, Germany). Frequencies and amplitudes of occlusal movements were recorded using PhysioMeter software. The applied occlusal force (visually presented via a bar graph) should be about 66% of the individual maximal occlusal force (visually presented as a horizontal line next to the bar) determined outside the MRI. Additionally, the same jaw movements were performed on the inserted stabilisation splint (splint task) and without any splint (no splint task) while no visual feedback was available.

All three tasks (tube task, splint task, no splint task) were performed respectively in a blocked design consisting of five resting blocks and four action blocks in alternating order each lasting 20 s (26 scans). During the action blocks, participants were asked to perform repetitive closing movements with 1 Hz visually paced by a white pulsating circle shown on a green background. During resting blocks, participants were instructed to keep their jaws in a relaxed position while the pulsating circle was displayed on a blue background.

The fMRI design was similar to that described before (Dammann et al. 2020; Ernst et al. 2019; Lickteig, Lotze, and Kordass 2013). Here, we are concentrating on the fMRI findings since clinical findings have already been published recently (Klepzig, Kordass, and Lotze 2024). Patients were placed in a supine position wearing hearing protection. Head movements were minimized by special cushions so that the mandibular movements could be carried out undisturbed. The whole examination started with the “tube task” while the order of the two following tasks (no splint, splint) was pseudorandomized to avoid habituation effects. Visual stimuli (pulsating circle, feedback of the occlusal force during the tube task) were presented via projector and a mirror attached to the head coil of the scanner. Prior to the examination, participants trained in occlusal movements outside the MRI scanner to get used to the procedure.

### 2.6 fMRI Scanning

fMRI scanning was performed with a 3Tesla Siemens Verio MRI (Erlangen, Germany) equipped with a 32‐channel head coil. Three different image types were acquired: (1) echo‐planar images (multi‐band EPI [CMRR]; multi‐band acceleration factor: 6, repetition time: 767 ms, echo time: 33.6 ms, flip angle: 54°, field of view: 210 × 210 mm^2^, 2.5 mm^3^ isotropic voxel size, 60 slices, no gap). (2) T1‐weighted high‐resolution structural whole head image using a three‐dimensional Magnetization Prepared Rapid Gradient Echo sequence (MPrage, repetition time: 1690 ms, echo time: 2.52ms, 176 sagittal slices, voxel size 1 × 1 × 1 mm).

### 2.7 Data Reduction and Preprocessing of fMRI Data

The Advanced Normalization Tools (ANTs, v2.3.5.dev212‐g44225) (Avants et al. 2011) were used to remove subject‐motion‐related artifacts by linearly registering each image of a fMRI time series to the first image of that series and to minimize susceptibility‐induced distortions by (non‐)linearly registering the average time series image to the T1w structural image. This was in turn (non‐)linearly registered to the MNI152 NLIN 6th gen. template space. The concatenated transformation of these registration steps was used to spatially normalize each fMRI time series into MNI space, and finally smooth the normalized time series using a Gaussian filter of 8 mm full‐width half maximum (FWHM).

### 2.8 Statistical Testing

The normal distribution of self‐reported data was examined using Kolmogorov–Smirnov tests. Demographic information and baseline characteristics (GCPS pain intensity, PCS score) of HCs and patients were compared using independent samples *t*‐tests. The distribution of self‐reported handedness and GCPS grades were examined using Fisher's exact tests. Since reported pain intensities (rest, movement) of HCs showed a significant deviation from normal distribution (right‐skewed; ps < 0.001) Mann–Whitney *U* tests were applied for comparisons with patients. As higher burdens were expected for patients compared to HCs one‐sided tests were used for comparing self‐reports (pain intensity, PCS). Performance measures assessed during the tube task (amplitudes and frequency of occlusions) were evaluated using homemade scripts running under MATLAB 7.4 and a repeated measures (rm) ANOVA with *Time* as within‐subjects variable (baseline, post1, post2) and Intervention (immediate, delayed) as between‐subjects variable to rule out differences interfering with therapeutic effects.

First‐level fMRI‐analysis: With respect to fMRI data for the first level effect event intervals within each trial were modelled as boxcar functions convolved with a canonical hemodynamic response function. Two different models were calculated: (1) each condition (tube, splint, no splint) and time (baseline, post1, post2) used for ANOVAs and (2) longitudinal comparisons for each condition (used for association analyses between clinical changes with changes in fMRI).

Second level analysis: Contrast images of each participant were then used for group statistics calculated as random‐effects analysis at the second level, which takes variance between participants into account. We calculated a full factorial model using rm ANOVA with the factors *Condition* (tube, splint, no splint) and *Time* (baseline, post1, post2) followed by *t*‐tests (Strauss et al. 2021). Since the *Condition* revealed relevant effects, we calculated another rmANOVA for comparing differences between conditions at baseline. We expected (1) an increase in anterior insula activation for occlusion without using the splint (no splint minus splint) and (2) an increase in occipito‐parietal areas for feedback and regulation of occlusal movements (tube minus splint).

We used an ROI approach correcting for multiple comparisons within the ROIs with *p* < 0.05 (family‐wise error; FWE). For the ROI analysis, we selected areas known to be involved in the processing of pain (anterior and posterior insula, S1, S2 and ACC) which we hypothesized should be decreasingly activated from baseline to post1 to post2 and should be associated with higher PC. In contrast, areas active during occlusal movements in healthy volunteers (Lotze et al. 2012; Mihai, von Bohlen und Halbach, and Lotze 2013) should be increasingly activated from baseline to post1 to post2 in TMD patients after successful intervention. These areas, processing sensorimotor control of occlusal movements, are the M1, the SPL and IPS, the SMA and anterior cerebellar hemispheres (Larsell's lobules 4–7). For anatomical masks, we used the ANATOMY Toolbox. If there were no suitable masks available (anterior/posterior insula, cerebellar hemispheres, anterior cingulum) we referred our analysis to the Neuromorphometrics atlas and the Automated Anatomical Labelling software (AAL).

Spearman's rank correlation analyses were performed for examining associations between self‐reported data (GCPS pain intensity, PCS score) and functional imaging activation for selected ROIs at baseline and for change scores (baseline−post2). According to our directional hypotheses, one‐tailed tests were applied. Here maximal beta values per ROI were extracted using in‐house scripts programmed in Matlab. Baseline beta values and their changes (baseline−post2) of both intervention groups were compared using independent samples *t* tests. For analyses of self‐reported data, comparisons of betas and correlations SPSS 22 (IBM Corporation, Armonk, NY, USA) was used, and the alpha level was set at 0.05. Imaging data analysis was realized using SPM12 (Wellcome Department of Imaging Neuroscience, London, UK). Based on a prior study on splint therapy in TMD patients with pain intensity after occlusion movements as an outcome measure (Lickteig, Lotze, and Kordass 2013) a relevant sample size of 16 patients was computed applying G*Power and a two‐sided paired *t*‐test with a Cohen's d of 0.992 (alpha = 0.05, power = 0.95).

## 3 Results

### 3.1 Comparisons at Baseline

#### 3.1.1 Characteristics of the Groups

Initially, TMD patients reported higher intensities of pain and reached higher chronic pain grades than HCs. Also, patients reached higher scores for PC than HCs (Table 1). Both intervention groups did not differ significantly regarding demographic data and intensity of self‐reported burdens.

**FIGURE 2:**
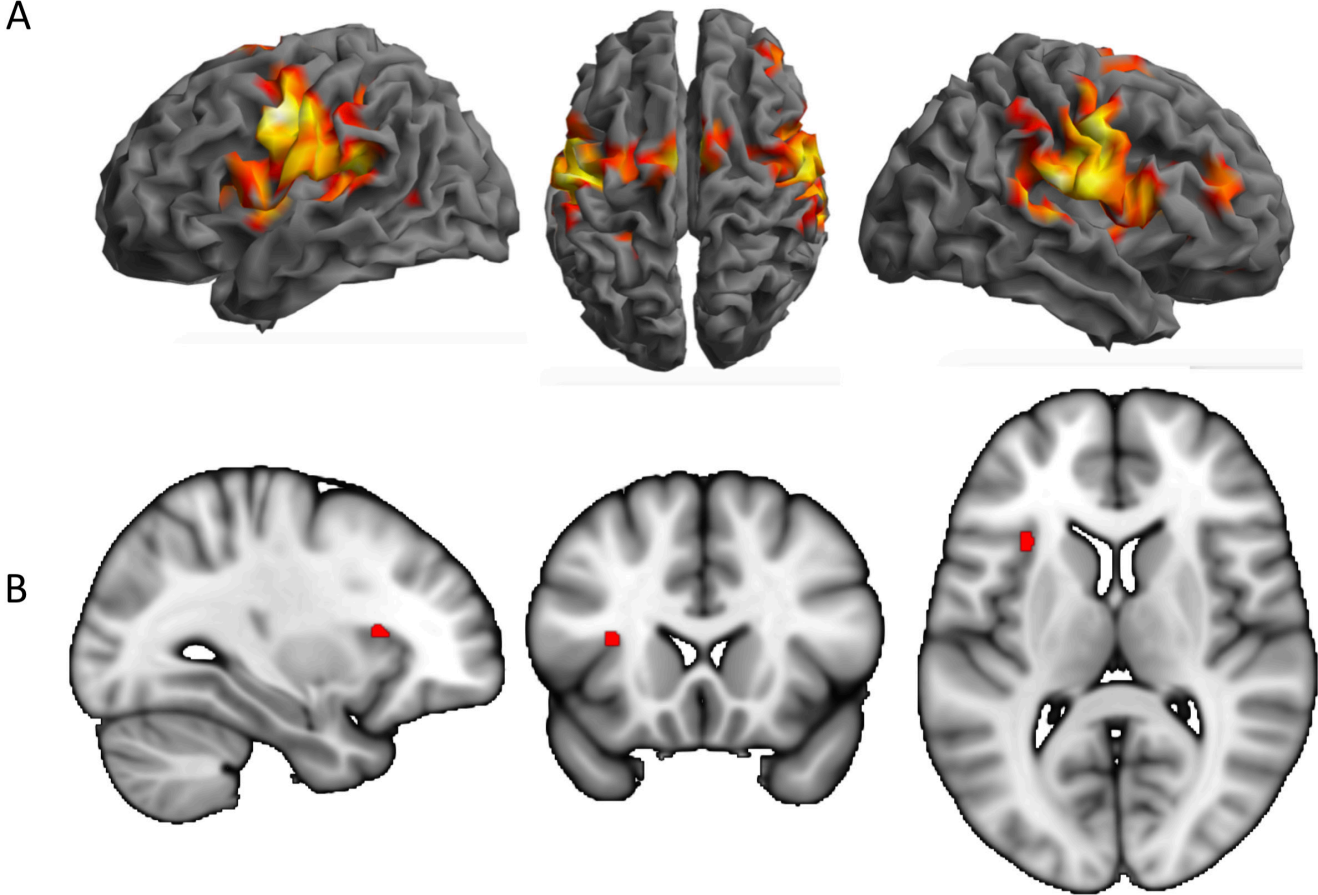
fMRI effects for TMD patients at the baseline. (A) Conjunction over all three conditions (*p*_FWE_ < 0.05) shows the well‐described occlusal representation in sensorimotor areas and the right prefrontal lobe (cerebellar representations not shown). (B) Differences between conditions (no splint minus splint) show the left anterior insula activation (thresholded for illustration purposes with *t* = 3).

fMRI showed a main effect on the *Condition* (Figure 2) which allowed for a more specific comparison at baseline. fMRI effects for all conditions revealed a group of regions comprising bilateral M1/S1, S2, insula, SMA, cingulate cortex, brainstem and cerebellar hemispheres (Figure 2A). Comparing between conditions (no splint minus splint) at baseline revealed an effect in the left anterior insula (*t* = 3.28; pone‐sided = 0.048; coordinates: −30, 19, 10) (Figure 2B). The visual control for modulating the intensity of maxillar pressure and frequency via the tube feedback task recruited additional resources in the occipital lobe (tube minus splint: left middle occipital gyrus: *t* = 6.43, *p*_FWE_whole brain_ = 0.001; coordinates: −40, −81, 40). Activations of the ACC and anterior insular cortex prior to therapy onset did not differ between intervention groups (ps > 0.1).

### 3.2 Correlation Analyses at Baseline

**FIGURE 3:**
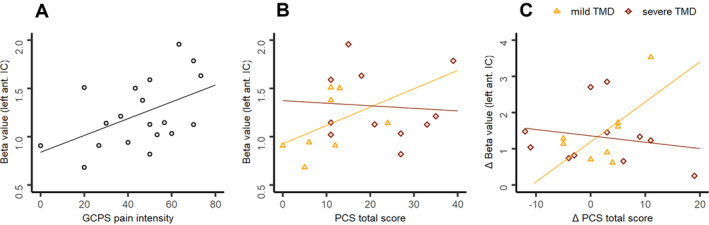
Plots show correlations between activation of the left anterior insula and self‐reports of GCPS pain intensity (A) and pain catastrophizing (PCS; B) for patients at baseline. Also, a correlation between changes over therapy (baseline−post2) in left anterior insula activation and pain catastrophizing is shown (C). A grouping regarding the GCPS grade is realized (B, C) with triangles indicating patients with mild TMD (GCPS grade < 2) and squares indicating patients with severe TMD (GCPS grade > 1). Regression lines are added. Abbreviations: Ant. = anterior, GCPS = Graded Chronic Pain Scale, PCS = Pain Catastrophizing Scale.

Activation in the left anterior insula during occlusal movements correlated positively with GCPS pain intensity in patients (*r*(19) = 0.459, *p* = 0.024; Figure 3A). No significant correlation with PC could be found (*r*(19) = 0.293, *p* = 0.112). However, when examining only those patients with mild TMD (GCPS grade < 2) we observed a trend for a positive association (*r*(8) = 0.575, *p* = 0.068), while such correlation was absent in patients with more severe TMD (GCPS > 2; *r*(11) = 0.009, *p* = 0.490; Figure 3B). Significant correlations with other brain regions could not be found.

### 3.3 Effects of Intervention

#### 3.3.1 fMRI Data

The ANOVA revealed significant effects for *Condition* and *Time* but no significant interaction between main factors. There were no significant (FWE‐whole brain and ROI approach) fMRI effects for the first 3 weeks of intervention (*n* = 20; between baseline and post1). However, at the end of the examination fMRI activation was reduced (baseline−post2) in the precuneus (*t* = 7.10, *p*_FWE_whole brain_ = 0.001, 591 voxel, coordinates: −5, −66, 40) and left angular gyrus (*t* = 6.90, *p*_FWE_whole brain_ = 0.001, 535 voxel, coordinates: −35, −66, 43). For the ROI analyses we found a significant decrease over time (baseline minus post2) in the right S1 (*t* = 5.04, *p*_FWE_ROI_ = 0.013, 64 voxel, coordinates: 20, −31, 60), the right S2 (*t* = 4.87, *p*_FWE_ROI_ = 0.026, 478 voxel, coordinates: 40, −14, 20) and the left insula (*t* = 4.71, *p*_FWE_ROI_ = 0.045, 11 voxel, coordinates: −62, −6, 6).

**FIGURE 4:**
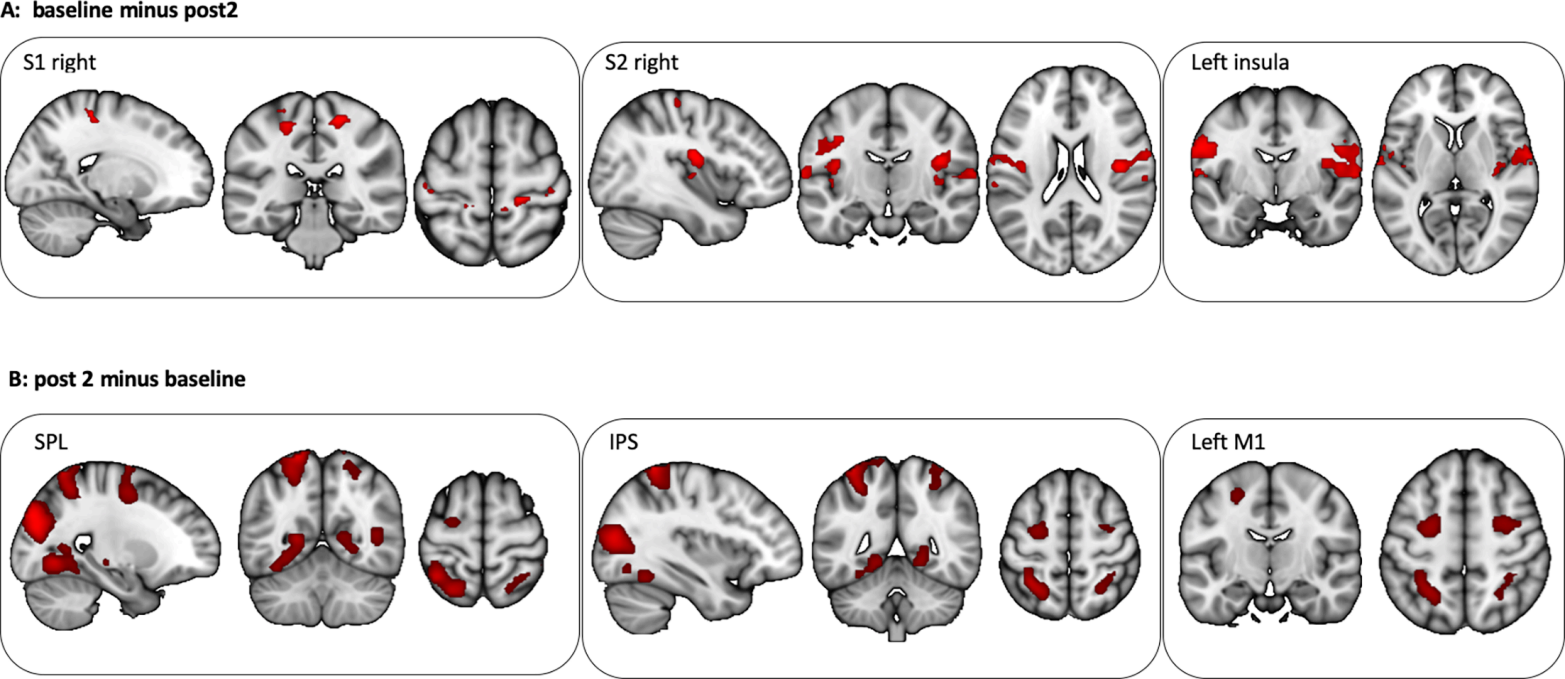
fMRI effects (ROI analyses) over time. Decrease (A) and increase (B) of fMRI activation during occlusion over intervention are shown. (A) S1 right: Coordinates: 20, −31, 60; S2 right: Coordinates: 40, −14, 20; left insula: Coordinates: −62, −6, 6. (B) SPL: Left: Coordinates: −18, −58, 70; IPS left: Coordinates: −35, −48, 56; left M1: Coordinates: −25, −8, 53.

Also, after the entire intervention (post2 minus baseline), TMD patients recruited higher fMRI activation in the left inferior occipital lobe (*t* = 8.70, *p*_FWE_whole brain_ = 0.001, 863 voxels, coordinates: −45, −74, 6) and left SPL (*t* = 7.12, *p*_FWE_whole brain_ = 0.001, 194 voxels, coordinates: −18, −58, 70). ROI analyses for the post2 minus baseline comparison revealed bilateral SPL (left: *t* = 7.12, *p*_FWE_ROI_ = 0.001, 338 voxels, coordinates: −18, −58, 70; right: *t* = 3.92, *p*_FWE_ROI_ = 0.025, 144 voxel, coordinates: 28, −54, 56), bilateral IPS (left: *t* = 4.31, *p*_FWE_ROI_ = 0.004, 10 voxel, coordinates: −35, −48, 56; right: *t* = 3.92, *p*_FWE_ROI_ = 0.014, 24 voxel, coordinates: 28, −54, 56) and left M1 (*t* = 4.80, *p*_FWE_ROI_ = 0.003, 46 voxel, coordinates: −25, −8, 53; Figure 4B). Analyses of occlusal performances during fMRI (tube task) showed no changes over time (*p* > 0.1), indicating consistent performance over the measurement time making longitudinal comparisons reasonable. Inserting age and duration of splint therapy as covariates for our analyses did not change the results. Decreases in activation of ACC and anterior insula over therapy were comparable in both intervention groups (ps > 0.1). No significant differences in occlusal performance between intervention groups and no significant interaction between Intervention type and Time could be stated.

### 3.4 Correlation Analyses Over Intervention

No significant association between reductions in PC and reductions in activation of any ROI during occlusal movements could be found (ps > 0.1). Again, when considering only patients with mild TMD a trending correlation between reductions in left anterior insula activation and catastrophizing could be shown (*r*(8) = 0.554, *p* = 0.077), which was not observable for patients with a more severe TMD (*r*(11) = −0.260, *p* = 0.221; Figure 3C).

## 4 Discussion

Recently, we showed in an RCT that wearing splints cannot only reduce pain intensity in TMD patients but also lower psychological distress such as PC (Klepzig, Kordass, and Lotze 2024). In the current work on the same patient cohort, we examined fMRI activity of critical brain sites during occlusal movements and associations with catastrophic thinking (at baseline and over therapy).

### 4.1 Effects at Baseline

At baseline, functional activation maps characteristic of occlusal movements (Mihai, von Bohlen und Halbach, and Lotze 2013) could be robustly determined across the three applied conditions allowing the usage of splints and online performance control via a tube which is highly relevant for the examination of intervention effects (i.e., to assure consistent occlusal force during the examinations). In line with previous work from our group (Lotze et al. 2012) we found between condition effects for occlusal movements in the left middle occipital gyrus probably related to the visual feedback and adjustment of movement performance (tube—splint) and in the left anterior insula underlying the functional effect of Michigan splints (no splint—splint).

Activation of the anterior insula has been associated with the subjective experience of interoceptive signals such as pain (Craig 2009). Interestingly, abnormalities in functional connectivity (Ichesco et al. 2012), grey matter volume (Younger et al. 2010) and functional activation of this structure were found in TMD patients compared to healthy controls (Roy et al. 2018) which probably reflects distorted pain processing in these cohorts (Yin et al. 2020). The positive associations between TMD pain intensity and left anterior insula activation during occlusal movements (at baseline) are consistent with these findings and suggest functional hyper‐activation in pain‐relevant structures which rises linearly with pain severity. Increased activation of the anterior insula could take effect as it might support pro‐nociceptive modulation resulting in more intense pain experience (Labrakakis 2023).

Pain processing was shown to be modulated by psychological processes such as increased attention and distraction (Apkarian et al. 2005). In fact, PC describes the recurrent and uncontrollable shift of the individual's attention to pain (Sullivan, Bishop, and Pivik 1995) which is finally associated with increased pain severity (Goubert, Crombez, and Van Damme 2004). Also, increased activation of the anterior insula was found during experimentally induced anticipation of pain (Ploghaus et al. 1999) which is a key component of PC (Sullivan et al. 2001). In contrast, distraction was found to lower the intensity of subjective pain experience and activation of the insula (Bantick et al. 2002). Hence, our finding of a positive correlation between left anterior insula activation during occlusion and the degree of catastrophic thinking about pain is plausible and confirms the relevance of PC for the development of TMD as previously suggested (Velly et al. 2011; Willassen et al. 2020). It has to be considered that the above relation was only found in patients with mild TMD symptomology. Interestingly, a similar correlation as shown here was also found in healthy controls, however, also restricted to (experimentally induced) mild pain (Seminowicz and Davis 2006).

### 4.2 Treatment Effects

Over therapy, functional activation was relevantly reduced for the left anterior insula and discriminative pain areas bilaterally (S1, S2), which partially confirms results reported for therapy with upper jaw splints in such patients before and could reflect the reduction of pain intensities over therapy (Ernst et al. 2019). Consistent with this we found a positive correlation between reductions in left anterior insula activation and PC. Similarly, a positive correlation between reductions in PC with reductions in functional connectivity between S1 and the left anterior insula after therapy was found in patients suffering from fibromyalgia (Lazaridou et al. 2017). In recent work (Dammann et al. 2020) we showed a similar correlation between changes concerning another type of dysfunctional thoughts (trait anxiety) and anterior insular activation (bilateral) over therapy which is in accordance with our data as trait anxiety is closely related to catastrophic thinking (Klepzig, Kordass, and Lotze 2024). Hence, addressing dysfunctional beliefs about pain appears highly relevant for improvements of symptoms in TMD patients as recently suggested in a general patient sample with pain of various types (Burns, Day, and Thorn 2012) while it also seems to be accompanied by changes in the neural representation of TMD pain.

We found correlations of therapeutic changes only in patients with less severe TMD. However, null correlations in patients with more severe TMD might be due to nonresponses towards splint therapy, which is relatively common in such cohorts (Litt and Porto 2013). Actually, increasing pain intensities (movement pain) over therapy were reported by about one third of the patients with more severe TMD (36.4%), while such worsening was reported only in one case of the patient group with mild TMD (12.5%). Areas associated with the physiological processing of occlusion (M1, SPL, IPS) showed increased activation after intervention which supports the results by He et al. (2016) and underlines the function of splint training for helping patients into more physiological activation patterns (Lotze et al. 2012).

Both intervention groups did not differ regarding decreases in fMRI activation over therapy (ACC, insular cortex). Hence, the advantages of a Michigan splint in reducing psychological distress and bodily arousal over a placebo splint as recently shown in the same cohort (Klepzig, Kordass, and Lotze 2024) must not be necessarily reflected by substantial differences in activation patterns during occlusion. An explanation might be that effects of the intervention type (immediate and delayed onset of Michigan therapy) on measures reported in the latter study were relatively subtle.

### 4.3 Limitations of the Study

The number of considered participants was relatively small as the work was realized as a unicenter study. As a result, the statistical power was not that high which might have hampered the detection of significant effects. Hence, future approaches should be realized as multicenter studies to increase the number of examined cases. It could be criticized that only women were considered in the study. However, men report lower symptom severity compared to women and are less likely to suffer from TMD in general (Schmid‐Schwap et al. 2013) which justifies an intervention study focused solely on women. Blinding of treatment allocation in patients might be hampered as two types of splints were applied in the delayed treatment group (placebo and Michigan) but only one splint in the immediate treatment group (Michigan). In fact, proper blinding is a frequent challenge in studies on splint therapies in TMD (Orzeszek et al. 2023). However, details of the two treatments were not given to the patients. Hence, they were unaware of the differences between them (i.e., a delay in the verum treatment). An additional issue might be an interaction of handedness and lateralization of the fMRI effect of occlusal movements (see Lotze et al. 2012). We here investigated almost only right‐handed participants with one participant showing a laterality index of 39.13 (Edinburg Handedness Inventory) indicating a more bilateral handedness than the others (recommended threshold for right handedness is 40; Oldfield 1971). Hence, our patient cohort can be more or less treated as right‐handed. Apart from this, handedness seems to be unrelated to chewing side and occlusion (Martinez‐Gomis et al. 2009). We, therefore, considered patients for analyses regardless of reported handedness as it is common practice in imaging and TMD (Festa et al. 2021; Nebel, Tommerdahl, and McGlone 2010; Peck et al. 2022; Vuong et al. 2020).

### 4.4 Conclusions

Our work showed that during occlusal movements, activations of structures relevant to affective pain processing were associated with TMD severity and PC. A decrease in activity of critical sites (e.g., insula) was observed during therapy and was partially associated with a reduction in PC. Hence, dysfunctional beliefs about pain appear critical for TMD while therapeutic effects (i.e., lowered catastrophizing) seem to be accompanied by changes in the cerebral representation of the occlusion process.

## Supporting information


**Figure S1** Flow chart depicting exclusions and drop outs of participants.

## Data Availability

The data that support the findings of this study are available from the corresponding author upon reasonable request.
