# Supplementary material for: Pain Catastrophizing and Functional Activation During Occlusion in TMD Patients—An Interventional Study
Source: Hum Brain Mapp. 2024 Oct 19;45(15):e70051. doi: 10.1002/hbm.70051 (PMC11489927; doi:10.1002/hbm.70051)
Supplement: Supplementary file 1 — Figure S1 Flow chart depicting exclusions and drop outs of participants. [file HBM-45-e70051-s001.pdf]

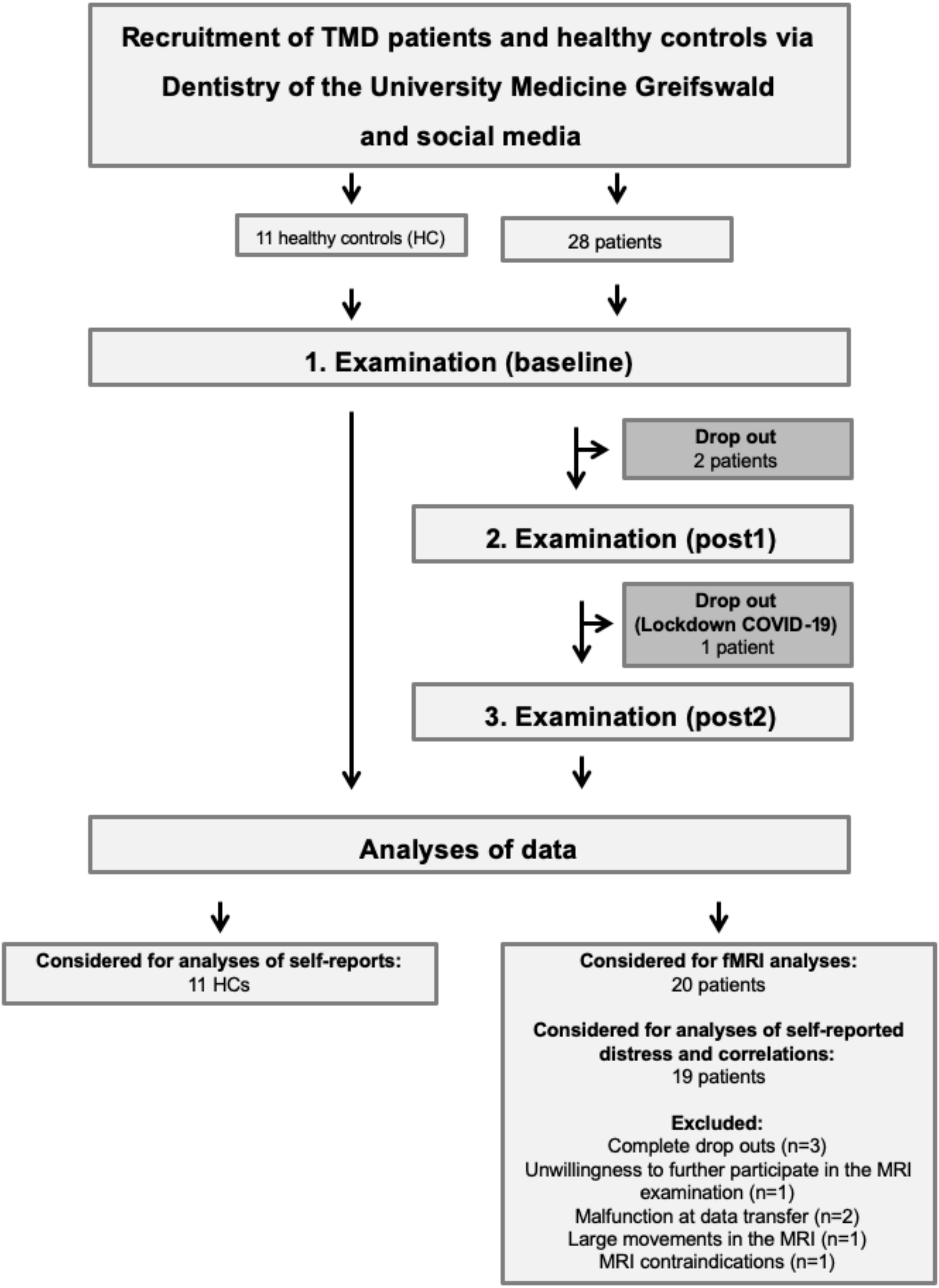
Recruitment of TMD patients and healthy controls via Dentistry of the University Medicine Greifswald and social media
11 healthy controls (HC)
28 patients
1. Examination (baseline)
Drop out
2 patients
2. Examination (post1)
Drop out
(Lockdown COVID-19)
1 patient
3. Examination (post2)
Analyses of data
Considered for analyses of self-reports:
11 HCs
Considered for fMRI analyses:
20 patients
Considered for analyses of self-reported distress and correlations:
19 patients
Excluded:
Complete drop outs (n=3)
Unwillingness to further participate in the MRI examination (n=1)
Malfunction at data transfer (n=2)
Large movements in the MRI (n=1)
MRI contraindications (n=1)
